# Cadmium Removal from Aqueous Systems Using* Opuntia albicarpa* L. Scheinvar as Biosorbent

**DOI:** 10.1155/2015/832571

**Published:** 2015-12-09

**Authors:** Rosa Icela Beltrán-Hernández, Gabriela Alejandra Vázquez-Rodríguez, Luis Felipe Juárez-Santillán, Ivan Martínez-Ugalde, Claudia Coronel-Olivares, Carlos Alexander Lucho-Constantino

**Affiliations:** ^1^Centro de Investigaciones Químicas, Universidad Autónoma del Estado de Hidalgo, Carretera Pachuca-Tulancingo Km 4.5, Ciudad Universitaria, 42067 Pachuca, HGO, Mexico; ^2^Programa de Ingeniería en Biotecnología, Universidad Politécnica de Pachuca, Ex-Hacienda de Santa Bárbara, Carretera Pachuca-Ciudad Sahagún Km 20, 43830 Zempoala, HGO, Mexico

## Abstract

The aim of this research was to investigate the use of a natural adsorbent like nopal (*Opuntia albicarpa* L. Scheinvar) for removing cadmium from aqueous solutions with low concentrations of this metal. Two treatments were applied to the cladodes: a dehydration to get dehydrated nopal (DHN) and heating up to 90°C to obtain a thermally treated nopal (TN). After examining the effect of various pH values (2–7), the capacity of each biosorbent was examined in batch sorption tests at different dosages (0, 500, 1000, 1500, 2000, and 3000 mg L^−1^). The results indicated that adsorption of cadmium to biomass of DHN and TN was highly dependent on pH and biosorbent dosage. The best removal of cadmium (53.3%, corresponding to *q*_*e*_ of 0.155 mg g^−1^) was obtained at pH 4.0 by using the TN sorbent. Infrared and Raman spectra confirmed that cadmium removal occurred via adsorption to –OH functional groups.

## 1. Introduction

Heavy metals receive world attention due to their long-term effects in the environment. Some of them, such as cadmium, are only found at trace level in the terrestrial crust. However, heavy metals are widely used in industrial processes and in commonly used goods, thereby increasing their presence and concentration in aquatic systems [1].

Cadmium has been classified into Group B1 as a probable human carcinogen by the US Environmental Protection Agency (EPA) and as a group I carcinogen by the International Agency for Research in Cancer (IARC) [2]. Consequently, World Health Organization, EPA, and the European Drinking Water Directive have established 0.005 mg L^−1^ as the maximum standard for Cd in water for municipal supply. In wastewater, EPA has set an upper limit of 2 mg L^−1^ in the Cd concentration before discharge to receiving water bodies [3].

Conventionally, heavy metals have been removed from wastewater by processes including chemical precipitation, coagulation/flocculation, flotation, ion exchange resins, absorption, and membrane filtration. However, the running costs of these processes are disadvantageous, as well as the generation of chemical sludges in the case of chemical precipitation [1, 4, 5].

Biosorption constitutes a low-cost and feasible method to remove heavy metals from aqueous streams. By using cheap and naturally abundant materials, this technology is well-suited for local and full-scale applications. Biosorbents such as living or dead microbial biomass, seaweeds, agricultural wastes, sawdust, and modified cellulosic materials have been studied for metal removal [1, 4–6].

In developing countries, the abundance and inexpensive nature of agricultural wastes make them a promising alternative to conventional chemical processes for heavy metal removal. Moreover, these natural sorbents yield biodegradable sludges and offer the possibility of metal recovering. Among the natural materials investigated with this purpose, nopal cladodes (*Opuntia* sp.) have been reported to have a high potential for removal of turbidity [7], ions from mine drainage [8], lead [9], and chromium [10]. Several biosorbents have been used for removing cadmium, and they have been shown to be a feasible alternative because high efficiencies and adsorption capacities (*q*_*e*_) have been obtained when elevated concentrations of cadmium (even as high as 1 g L^−1^) were tested [1].

This paper reports the removal of low concentrations of cadmium from aqueous media by using cladodes of* Opuntia albicarpa* L. Scheinvar. The objective of testing low concentrations was to simulate more common conditions, that is, cadmium levels comparable to the upper limit of 2 mg L^−1^ established for wastewater by EPA [3]. Two biosorbent preparation methods were tested in terms of removal efficiency. Additionally, functional groups interacting with cadmium were identified by using FTIR and Raman spectroscopy.

## 2. Materials and Methods

### 2.1. Cadmium Solutions

A stock cadmium solution (10000 mg Cd^2+^ L^−1^) was prepared by dissolving 1 g of metallic cadmium (99.9% of purity, Sigma Aldrich) in 20 mL of concentrated HNO_3_. The solution volume was completed up to 100 mL with deionized water (Milli Q Plus Millipore Columns Systems). Cadmium solutions (1.6 ± 0.02 mg Cd^2+^ L^−1^) used in biosorption studies were made by appropriate dilution of the stock solution with deionized water.

### 2.2. Natural Materials

Nopal specimens of* Opuntia albicarpa* L. Scheinvar were collected from Zempoala (Hidalgo, Mexico), and only one-year aged cladodes were retained for the study. Prior to treatment, cladodes were washed with deionized water and cut in 3 cm × 1 cm segments of about 10 g each.

### 2.3. Biosorbent Preparation

Biosorbents were obtained following two different treatments. Dehydrated nopal (DHN) was prepared by drying cladode segments at 70°C for 5 h. In this way, a reduction of 82.7% of the total water content could be achieved. Next, cladodes were ground using an electric blender (Waring, USA) and sieved through 250–600 *μ*m mesh screens (Tyler test sieves #60 and #30, resp.).

Thermally treated nopal (TN) was prepared by heating segments of cladodes (200 g in total) with 500 mL of water until boiling occurred (approximately to 90°C at about 2000 meters above sea level). Then, the bulk mixture was liquefied and dehydrated at 70°C for 24 h. Dried nopal was ground and sieved to obtain a particle size lower than 600 *μ*m.

### 2.4. Biosorption Tests

Batch sorption tests were carried out in triplicate on a standard jar test apparatus (Quimipura, Mexico). Each jar was filled with 500 mL of cadmium solution (of about 2 mg Cd^+2^ L^−1^) and then stirred at 150 rpm for 1 min. Stirring speed was then lowered to 85 rpm for 4 min and after set to 20 rpm during 10 min. Finally, samples were allowed to settle for 30 min [11]. The sorbent was recovered by filtration of the settled material through Whatman 42 filter paper.

To assess cadmium removal, supernatant samples (taken before and after a given treatment) were digested in a microwave oven (MARSX-5) by following a standard technique [12]. Subsequently, samples were analyzed in an Atomic Absorption Spectrophotometer (AAS, Varian 800).

The effect of pH on cadmium removal was studied at different pH values (2–7) of the cadmium solution and with 500 mg L^−1^ of DHN. pH was adjusted by adding 1.0 M HCl or 1.0 M NaOH. The effect of the biosorbent dosage was studied at five doses (500, 1000, 1500, 2000, and 3000 mg L^−1^) of both biosorbents.

### 2.5. Modeling of Biosorption

Percentages of cadmium removal were adjusted to the Chapman sigmoidal expression:

(1) $$\begin{array}{c} \text{\% Cd removal}=a{\left(\;1-e^{-b\cdot m}\;\right)}^{c}, \end{array}$$

where *a* is the maximum percentage of Cd removal, *m* is the dosage of biosorbent, and *b* and *c* are empirical constants. Curve fitting was carried out using SigmaPlot for Windows version 12.0 (Systat Software, Inc.).

### 2.6. FTIR and Raman Spectroscopy Analyses

Before and after being used in the jar tests, biosorbents were screened by Fourier transform infrared (FTIR) spectroscopy (Perkin Elmer Spectrum GX) using the KBr pellet method.

Raman spectra were obtained on a Perkin Elmer Spectrum GX, equipped with a Raman module attachment, a Nd^3+^ laser operating at 1064 nm in the near infrared, and a InGaAs detector cooled with liquid N_2_. The spectral resolution used was 4 cm^−1^. Samples of biosorbents were prepared in the same way (KBr pellet) as for FTIR analyses. Likewise, biosorbents were analyzed before and after being used in jar tests.

## 3. Results and Discussion

### 3.1. Effect of pH on Biosorption

**Figure 1:**
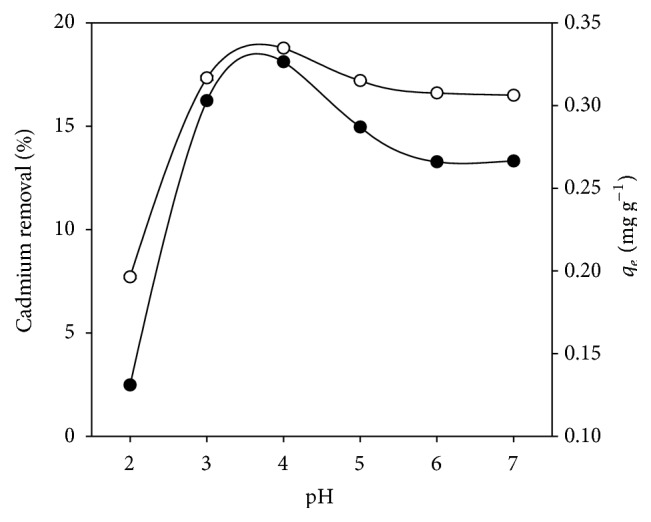
Effect of pH on cadmium removal by dehydrated nopal (DHN). (-○-) Percentage of cadmium removal; (-●-) *q*_*e*_, adsorption capacity [mg Cd adsorbed g^−1^ biosorbent].

Since pH of aqueous media is an important parameter controlling adsorption processes of heavy metals [16], the effectiveness of DHN biosorbent was examined at different initial pH values (2–7). The initial cadmium contents comprised between 1.593 and 1.806 mg L^−1^. After the jar tests, concentrations of cadmium comprising between 1.32 and 1.63 mg L^−1^ were measured.  Figure 1 shows the results in terms of cadmium removal percentages and adsorption capacity (*q*_*e*_, mg Cd adsorbed g^−1^ biosorbent). On the one hand, the lower cadmium removal observed at pH 2 suggests a competition between Cd^2+^ and H^+^ for the adsorption sites. On the other hand, the greatest cadmium removal (18.5%, corresponding to *q*_*e*_ of 0.327 mg g^−1^) was registered at pH 4. Barrera et al. [8] reported that the uptake of Cr(III) and Cr(VI) in acid mine drainage by* Opuntia* sp. was maximum at pH 4 (99 and 77%, resp.). Similarly, a pH range of 3–5 allowed a higher amount of Pb^2+^ to be adsorbed by* Opuntia streptacantha* [9]. Fernández-López et al. [10] also found that the adsorption of hexavalent chromium by* Opuntia* cladodes is pH dependent and maximum at pH 2. Consequently, an acidic pH value of 4 was used in further testing of the biosorbents.

### 3.2. Effect of Biosorbent Dosage on Cadmium Removal

**Figure 2:**
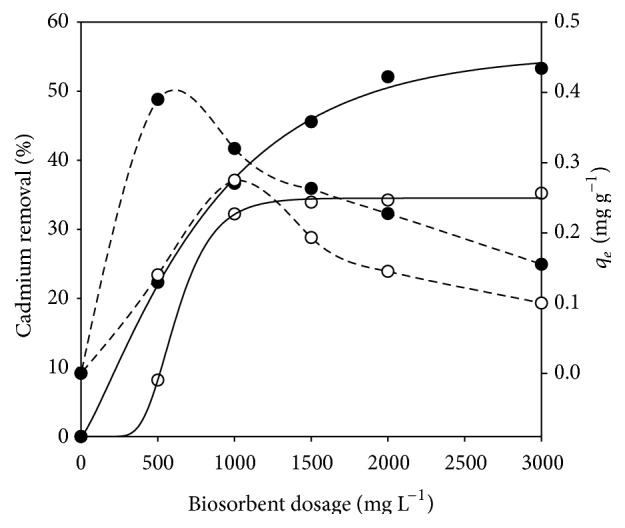
Effect of biosorbent dosage on cadmium removal. (-○-) DHN; (-●-) TN. The continuous lines represent the modeling of the percentages of cadmium removal. The dashed lines represent the progression of *q*_*e*_, adsorption capacity [mg Cd adsorbed g^−1^ biosorbent].

The effect of varying the dosage of both biosorbents (DHN and TN) from 500 to 3000 mg L^−1^ at pH 4 is shown in Figure 2. For DHN tests, the initial cadmium concentration was 1.69 mg L^−1^. At the end of jar tests, cadmium contents comprised between 1.09 and 1.55 mg L^−1^. The cadmium uptake increased from 8.1 to 35.2% when the dose of sorbent was raised from 500 to 1000 mg L^−1^. The measured value of *q*_*e*_ also varied (from 0.14 to 0.275 mg g^−1^) along with the increase of the biosorbent dose. Further increases of the dose of biosorbent led to a diminution of *q*_*e*_ (equivalent to 0.1 mg g^−1^ for a dosage of 3000 mg L^−1^). Barka et al. [15] tested dehydrated* Opuntia ficus* for removing high contents of cadmium (30–300 mg L^−1^), and they obtained efficiencies comprised between 10.49 and 36.71% by using 500–4000 mg L^−1^ of biosorbent, respectively. Apparently, the use of dehydrated* Opuntia* sp. at these doses leads to low-to-moderate removal efficiencies to be obtained. In fact, biosorbents used for removing low cadmium concentrations (1–10 mg L^−1^) generally bring about low-to-moderate removal efficiencies [1, 13–15], which is in agreement with our results (Table 1).

For TN tests, the initial cadmium concentration was 1.74 mg L^−1^. After the jar tests, cadmium contents were comprised between 0.81 and 1.35 mg L^−1^. The increase of biosorbent dosage from 500 to 2000 mg L^−1^ increased cadmium uptake from 22.3 to 53.3% and diminished *q*_*e*_ from 0.39 to 0.155 mg g^−1^ (Figure 2). Presumably, enhancement of cadmium removal at higher biosorbent doses is due to an increase in adsorption sites. However, further increasing of biosorbent does not appear to be significant for improving cadmium removal.

The results obtained by simulation of the Chapman equation are also shown in Figure 2. The model appears to depict the experimental data adequately, because the Pearson correlation coefficients (*r* ^2^) were 0.998 and 0.999 for TN and DHN, respectively. The *a* value of (1), corresponding to the maximum percentage of Cd removal, was 55.4 and 34.5, respectively, and so the adsorption capacity of the biosorbent was enhanced by the thermal treatment. The *b* and *c* values were 0.0013 and 1.287 for TN results, respectively. For DHN, *b* and *c* were found to be 0.006 and 27.92, respectively.

### 3.3. FTIR Spectra of Biosorbents

The main functional groups involved in biosorption are binding groups located on the surface of cell wall, as carboxyl, sulfonate, phosphate, amino, amide, and imidazole [16]. Infrared spectroscopy allows the identification of these moieties, as well as the detection of changes in their vibrational modes due to bond formation.

**Figure 3:**
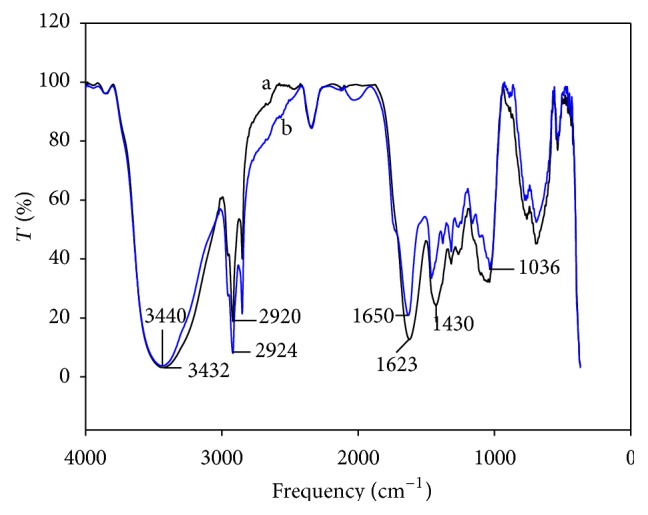
FTIR spectra of the dehydrated biosorbent (a) before and (b) after the jar tests.

Figure 3 shows FTIR spectra of DHN before and after contact with a Cd solution at pH 4. Before exposition to cadmium, the spectrum of DHN sorbent showed tension bands at 3432 and 2924 cm^−1^, corresponding to O–C and C–H bonds, respectively. C=O tension bands were observed too, but at a lower frequency (1650 cm^−1^ instead 1700 cm^−1^). This can arise from mutarotation changes of ketomoieties into aldehyde or alcohol groups in biosorbent sugars, which are likely to diminish the tension in the C=O bond. The band found at 1623 cm^−1^ is assigned to C=O stretching in amide groups. Finally, in the range of 1318–1036 cm^−1^, the bands pointed to C–N and C–O stretching or deformation vibrations of –CH, –OH, or –NH bonds.  Table 2 summarizes the association between infrared adsorption frequencies and functional groups observed in FTIR spectra of biosorbents (DHN and TN), which could be responsible for cadmium removal.

After being in contact with the Cd solution, a light modification in the 3432–3440 cm^−1^ region (assigned to –OH and –NH bond stretching) was noticed. The bands in the 1623–1036 cm^−1^ range also showed a small displacement. These changes in the spectra could indicate Cd sorption to the DHN. Likewise, Gupta and Rastogi [17] showed Cd adsorption when* Oedogonium* sp. was used as biosorbent through shifts of the IR bands at 1082, 1650, and 2924 cm^−1^.

**Figure 4:**
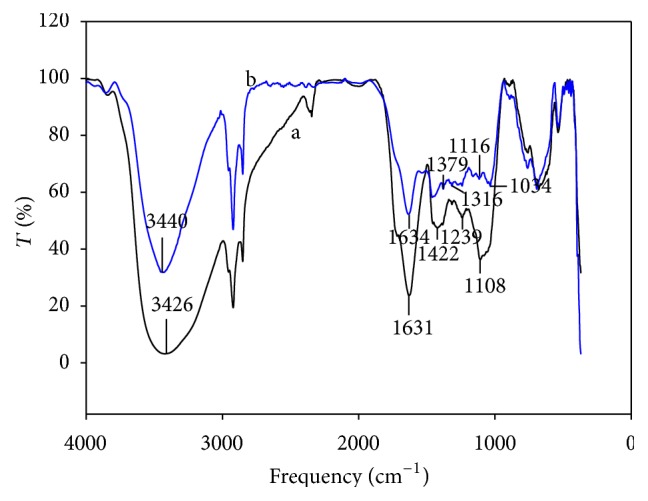
FTIR spectra of the thermally treated nopal (a) before and (b) after the jar tests.

**Figure 5:**
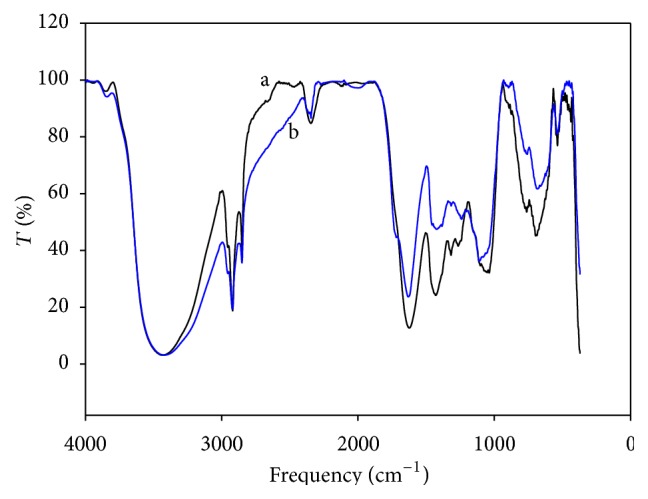
Comparison of FTIR spectra of the dehydrated biosorbent (a) before and (b) after the jar tests.

Thermally treated nopal showed a strong interaction with cadmium (Figure 4). After adsorption tests, bands at 2900–3600 cm^−1^ region (assigned to –OH and –NH groups) exhibited a lower intensity, possibly due to presence of Cd in these sites. Band intensity in the 1634–1034 cm^−1^ region also diminished after the exposure to Cd. In Figure 5, functional groups of the thermally treated nopal are compared against those of the dehydrated biosorbent. In a general manner, the TN sorbent produced bands of lower intensities than those of dehydrated nopal, even though the later allowed a higher cadmium removal.

### 3.4. Raman Spectra of Biosorbents

As FTIR spectra did not indicate the presence of formal bonds, Raman spectroscopy was used to characterize superficial interactions between biosorbents and Cd.

**Figure 6:**
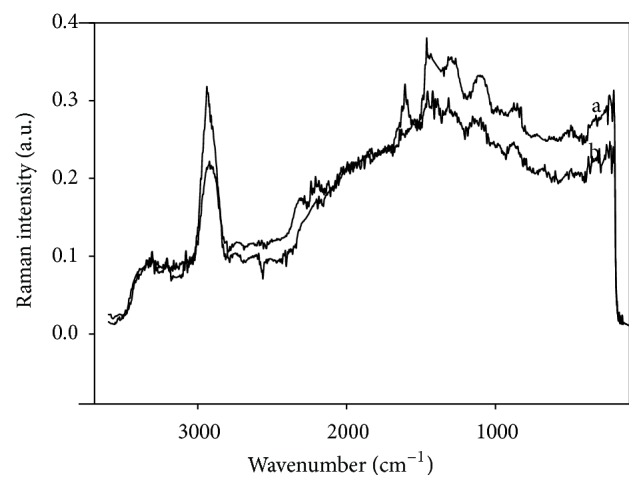
Raman spectra of the dehydrated biosorbent (a) before and (b) after the jar tests.

Figure 6 shows the spectrum of DHN biosorbent before and after being in contact with Cd. Bands assigned to C=O (1690 cm^−1^), COO–H (1690 and 1615 cm^−1^, with scissor vibration), and C–C (1558–1535 cm^−1^) groups were detected, as well as an intense C–H band at 3045–3035 cm^−1^. The band at 3000 cm^−1^ is probably due to –OH moieties and water [18]. Also, less-intense bands at 1088–1062 cm^−1^ (in-plane) and at 1033–1027 cm^−1^ (out-of-plane, deformation) were observed. Such bands, although less intense, were also present after adsorption tests, indicating a weak adsorption of Cd on DHN sorbent. The rest of the bands were superimposed and less defined than that of the C–H bond, which is understandably the main functional group in compounds present in* Opuntia albicarpa* L. Scheinvar.

**Figure 7:**
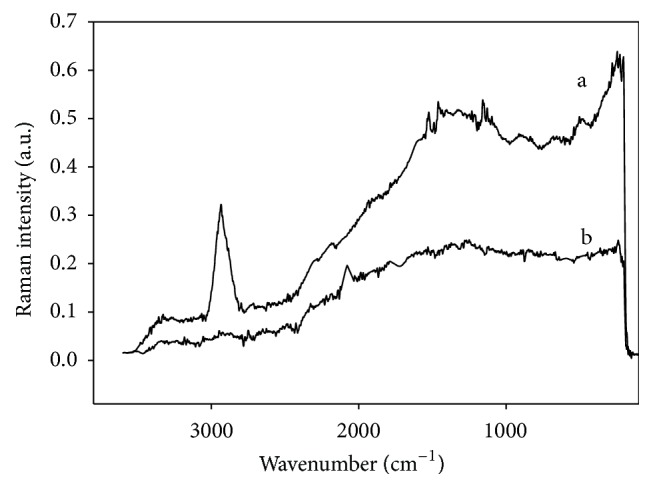
Raman spectra of the thermally treated biosorbent (a) before and (b) after the jar tests.

Treated TN showed the same bands as DHN when sorbents were analyzed by Raman spectroscopy prior to adsorption tests (Figure 7). However, after being exposed to Cd solution, these bands disappeared in TN. The lack of bond vibration indicated that cadmium was retained only on the biosorbent surface.

## 4. Conclusions

Thermally treated* Opuntia albicarpa* L. Scheinvar biomass presented the best removal of cadmium in biosorption studies. Metal adsorption was highly dependent on the pH and biosorbent dosage. IR and Raman spectra confirmed that the removal of cadmium occurred via adsorption and showed the interaction between cadmium and some functional groups (O–H, N–H, N–C=O, C–O, and C–N). Since nopal is a natural material widely distributed around the world, and the preparation of the TN biosorbent is easy and inexpensive, it could constitute an adequate metal removal technology for developing countries.

## Figures and Tables

**Table 1:** Cadmium removal by biosorption.

Bioadsorbent	[Cd]_i_ (mg L^−1^)	Sorption capacity^a^ (mg/g) or removal efficiency^b^ (%)	Experimental conditions	Reference
Rice husk	11.24	26.73^a^	[B] = 500 mg L^−1^ *T* = 25°C pH = 2.0–6.0	[13]

Olive tree pruning waste	1–10	36.6^a^	[B] = 100 mg L^−1^ pH = 5.5 *T* = 21 ± 0.4°C *t* = 120 min	[14]

Dried cladodes (*Opuntia ficus*)	30–300	12.07–30.42^a^ 10.5–36.7^b^	[B] = 500–4000 mg L^−1^ pH = 2.3–6.5 *T* = 25–60°C	[15]

Dehydrated nopal (DHN) of *Opuntia albicarpa *L. Scheinvar	1.6	0.14–0.275^a^ 8.1–35.2^b^	[B] = 500–1000 mg L^−1^ pH 4.0	This study

Thermally treated nopal (TN) *Opuntia albicarpa *L. Scheinvar	1.6	0.39–0.23^a^ 22.3–53.3^b^	[B] = 500–2000 mg L^−1^ pH 4.0	This study

[Cd]_i_: initial cadmium concentration, [B]: dosage of biosorbent.

**Table 2:** Functional groups present in treated *Opuntia albicarpa *L. Scheinvar and their corresponding infrared absorption frequencies.

Frequency (cm^−1^)	Assignment
3600	N–H amines groups
3428	Hydroxyl group
2850	C–H stretching
1623	C=O stretching of COOH
1414	Symmetric bending of CH_3_
1317	C–N groups
1050	C–O groups
